# Assessment of Cu and As in Wheat (*Triticum aestivum* L.) and Arable Land in the Vicinity of Bor (Serbia): Implications for Food Safety and Human Health

**DOI:** 10.3390/plants15040631

**Published:** 2026-02-16

**Authors:** Danijela Simonović, Daniel Kržanović, Renata Kovačević, Mirjana Šteharnik, Sunčica Stanković, Danijela Urošević, Vesna Krstić

**Affiliations:** 1 Mining and Metallurgy Institute Bor, Alberta Ajnštajna 1, 19210 Bor, Serbia; danijela.simonovic@irmbor.co.rs (D.S.); daniel.krzanovic@irmbor.co.rs (D.K.); renata.kovacevic@irmbor.co.rs (R.K.); mirjana.steharnik@irmbor.co.rs (M.Š.); daniela.urosevic@irmbor.co.rs (D.U.); 2 Faculty of Management, Metropolitan University, 11158 Belgrade, Serbia; suncica.stankovic@metropolitan.ac.rs; 3 Technical Faculty Bor, University of Belgrade, Vojske Jugoslavije 12, 19210 Bor, Serbia

**Keywords:** Cu and As with accompanying elements, arable land, ICP-MS, ecological indices, bioaccumulation of wheat, food safety, MANOVA

## Abstract

Mining exploitation and copper smelting in Bor (Serbia) have led to long-term environmental pollution with toxic metals, primarily copper (Cu) and arsenic (As). The aim of this research was to assess the contamination of arable land and the bioaccumulation of metals in wheat (*Triticum aestivum* L.), to determine significant differences in copper and arsenic concentrations between the soil and specific wheat tissues across six locations, and to evaluate environmental and health risks in agricultural areas around the Zijin Copper Mine, Serbia. Sampling was carried out at six locations (Brezonik, Veliki Krivelj, Oštrelj, Slatina, Zlot, and Gornjane; L1–L6, respectively). Analyses of soil and wheat to determine toxic elements were performed using the ICP-MS method, while contamination was assessed using descriptive statistics and a combination of several indices (CV, Igeo, EF, CF, Er, RI, PLI, BAF, TF, and HRA). In addition to Cu and As, accompanying elements (Fe and Al) were also included in the analysis, due to their importance as indicators of geogenic and anthropogenic origin. The analysis of the distribution within the root, stem, leaf, and grain of wheat enabled the assessment of bioaccumulation (BAF and TR) and implications for food safety (HRA). The results showed that concentrations of Cu and As at several locations significantly exceed the regulatory limit values, with Slatina-L4 and Oštrelj-L3 identified as the most polluted areas, while Gornjane-L6 can be considered a reference location with minimal risk. Background values were taken from location L6, considered a reference site due to the absence of direct mining and industrial influence (B_Cu_—20 mg/kg, B_As_—10 mg/kg, and B_ref·Al_—33,300 mg/kg). The MANOVA analysis revealed statistically significant differences in copper and arsenic concentrations between the soil and various wheat tissues, with the effect being more pronounced for arsenic. The integrated analysis of indices (RI and PLI) confirmed the pronounced anthropogenic impact and location-specific risks, emphasizing the need for continuous monitoring, locally adapted remediation strategies, and sustainable land management.

## 1. Introduction

Mining and metallurgical activities represent one of the main anthropogenic sources of environmental pollution, especially in regions with intensive copper production. The Bor mining and metallurgical basin in eastern Serbia is recognized as one of the most polluted areas in Southeastern Europe, with long-term emissions of toxic metals and metalloids, primarily copper (Cu) and arsenic (As), originating from smelters, flotation tailings, and landfills [1,2,3]. Numerous studies have documented elevated concentrations of elements in soil, in the water, plants, and air in Bor, confirming serious ecological degradation and potential risks to human health [4,5,6].

Toxic metals and metalloids released by mining and metallurgical processes accumulate in agricultural soils, leading to the contamination of crops and their entry into the food chain [7,8]. Research in Bor, as well as in other mining regions around the world, emphasizes the importance of assessing soil contamination and bioaccumulation in plants for evaluating environmental and health risks [9,10,11]. It was recently confirmed that agricultural soils in the central and western regions of Serbia show elevated concentrations of heavy metals, indicating a widespread problem [12].

Plants develop various physiological and biochemical mechanisms to mitigate the toxicity of heavy metals, including retention in the roots, restricted translocation to aboveground organs, binding of metals in cell walls, and complexation with organic ligands [13]. In addition, elements such as silicon (Si), iron (Fe), and aluminum (Al) may contribute to alleviating toxic effects through mechanisms of competition and stabilization in the soil [14].

Previous research has mainly focused on toxic elements such as Cu, As, Pb, Cd, and Zn. Integrating toxic and essential elements into contamination assessments enables a more comprehensive understanding of agroecological stability and food safety.

The novelty of this work is reflected in the integrated approach, which integrates quantitative indices of soil pollution (CV, Igeo, EF, CF, Er, IR, and PLI) with bioaccumulation in wheat (*Triticum aestivum* L.) (BAF), one of the most important food crops in Serbia, as well as in combination with health indices (HRA, including HQ and HI) applied at specific locations. This makes this work one of the few studies in Southeast Europe that simultaneously considers ecological indices, bioaccumulation, and implications for food security, providing a basis for the development of sustainable land management strategies in mining regions.

Wheat (*Triticum aestivum* L.) was selected as the plant species traditionally cultivated in the Bor region of eastern Serbia on agricultural land located in close proximity to mining and metallurgical facilities and used for both human and animal consumption.

The goals of this research were: (i) to assess the degree of contamination of arable land and wheat in the vicinity of the Zijin Copper mine (Bor, Serbia) using the ICP-MS method to determine the concentration of elements; (ii) to determine the significance of differences in Cu and As concentrations between soils from different locations and various parts of the wheat plant system; (iii) to quantify pollution using a combination of indices (CV, Igeo, EF, CF, Er, IR, and PLI) to identify location-specific risks; (iv) to investigate the distribution of Cu and As in different parts of wheat (root, stem, leaf, and grain); (v) to separate geogenic and anthropogenic sources of contamination through the analysis of essential macro- and microelements; (vi) to assess the implications for agroecological stability and food security in the mining region of Bor; and (vii) to propose directions for continuous monitoring, locally adapted remediation strategies, and sustainable land management.

## 2. Results

### 2.1. Descriptive Statistics

The results of descriptive statistics, shown in Table 1, indicate a pronounced spatial heterogeneity of copper (Cu) and arsenic (As) concentrations in arable land in the vicinity of Bor.

Descriptive statistics (Table 1) show that the highest mean was recorded for Cu at location L4—Slatina (568.60 ± 7.66). At the same location, the highest mean for As was also observed (63.05 ± 0.81). The coefficient of variation (CV) values are very low (1.35% for Cu and 1.29% for As), which suggests stable and reproducible results. The lowest mean values for both elements were recorded at location L6—Gornjane, i.e., 8.71 ± 0.14 for As and 20.28 ± 0.01 for Cu. The CV for copper is extremely low (0.03%), almost negligible, which indicates a very homogeneous copper sample at this location. These results lead to the conclusion that location L4 is the most contaminated site with both copper and arsenic and therefore requires special attention due to the high concentrations of these toxic elements. Conversely, location L6 is the least contaminated. At location L1—Brezonik, moderate concentrations of Cu (about 217 mg/kg) and As (about 15 mg/kg) were recorded, with similar CV values for both metals (3.92% for Cu and 3.46% for As). Low concentrations of Cu (about 75 mg/kg) and As (slightly less than 10 mg/kg) were recorded at location L2—Veliki Krivelj, where the CV for As is slightly higher (4.13%), indicating greater relative dispersion. A high concentration of Cu (about 377 mg/kg) and moderate As (slightly more than 28 mg/kg), along with very low CV values for both elements (<1.5%), were recorded at location L3—Oštrelj, which indicates exceptional consistency of measurements. Locations L3 and L4 show the lowest CV values, suggesting that contamination is evenly distributed or that sampling was more homogeneous. A low concentration of Cu (slightly less than 61 mg/kg) but a high As concentration (almost 32 mg/kg) was recorded at location L5—Zlot. It was observed that the CV for As is higher (3.18%) than for Cu (1.9%), which may reflect sample heterogeneity in terms of As at this site. In most cases, the CV is below 5%, indicating good measurement precision and sample homogeneity. Detailed information on the maximum and minimum values of both elements at each location is provided in Table 1.

Analysis of Cu and As concentrations in wheat (Table 2) shows that the values differ by plant organs and locations and are lower compared to the results in soil. The root remains the main reservoir of contaminants (Cu up to 112 mg/kg; As up to 8.86 mg/kg at L4), while the grain shows relatively low values but still indicates the presence of Cu and As at contaminated locations.

### 2.2. Determination of Statistically Significant Differences in Cu and As Concentrations

Preliminary analysis using the Shapiro–Wilk test indicated that the data did not follow a normal distribution, which required logarithmic transformation prior to further statistical processing. Before applying the MANOVA method, Box’s M test was conducted to verify the assumption of homogeneity of covariance matrices. The test results showed that the assumption of homogeneity was not violated (Box’s M test, *p* = 0.186 > 0.001). The results of the one-way multivariate analysis of variance (Table 3) demonstrated that the Soil_Plant_Part factor had a statistically significant multivariate effect on the set of dependent variables. All applied multivariate criteria (Pillai’s Trace, Wilks’ Lambda, Hotelling’s Trace, and Roy’s Largest Root) indicated a statistically significant effect (*p* < 0.001). Based on Wilks’ Lambda (Λ = 0.102; F(8.48) = 12.824; *p* < 0.001), it can be concluded that there is a statistically significant difference between the groups defined by the Soil_Plant_Part factor. The values of partial eta squared (ηp^2^ = 0.681) [15] indicate a large effect, meaning that the Soil_Plant_Part factor explains a substantial proportion of the variance in the dependent variables.

For the continuation of the MANOVA analysis, it was necessary to conduct a series of univariate ANOVA tests for each dependent variable. The risk value (α) assigned for performing multiple ANOVA tests had to be divided by the number of tests conducted and then compared with the corresponding *p*-value.

In this case, the assigned value of 0.05 was converted to 0.025 (0.05/2). Based on the results presented in Table 4, for the dependent variable logCu, differences between soil and different parts of the plant system were statistically significant (F(4,25) = 13.618; *p* < 0.001), with partial eta squared (ηp^2^ = 0.685) indicating a large effect. For the dependent variable logAs, the differences were also statistically significant (F(4,25) = 42.898; *p* < 0.001), and partial eta squared (ηp^2^ = 0.873) showed a large effect size, meaning that different soils and plant parts explained a greater proportion of the variance in arsenic concentrations compared to copper.

After confirming statistically significant differences with one-way ANOVA, post-hoc testing was performed using Tukey’s HSD test to identify differences between specific parts of the plant system. The results of the post-hoc Tukey HSD test (Table 5) showed that copper concentrations in soil were significantly higher compared to leaf (Mean Difference (MD) = 1.04; *p* = 0.001; 95% CI: 0.37–1.70), stem (MD = 1.44; *p* = 0.0001; 95% CI: 0.77–2.10), and grain (MD = 1.31; *p* = 0.0001; 95% CI: 0.65–1.98), while the difference between soil and root was not significant (MD = 0.58; *p* = 0.110). Root exhibited significantly higher copper concentrations compared to stem (MD = 0.86; *p* = 0.007; 95% CI: 0.20–1.52) and grain (MD = 0.74; *p* = 0.024; 95% CI: 0.07–1.40), whereas differences between root and leaf were not statistically significant (*p* = 0.277). No significant differences in copper concentrations were found among leaf, stem, and grain (*p* > 0.05).

For arsenic, more pronounced differences were observed. Soil had significantly higher arsenic concentrations compared to root (MD = 0.82; *p* = 0.005; 95% CI: 0.20–1.43), leaf (MD = 1.45; *p* = 0.0001; 95% CI: 0.83–2.06), stem (MD = 1.94; *p* = 0.0001; 95% CI: 1.32–2.55), and grain (MD = 2.50; *p* = 0.0001; 95% CI: 1.88–3.11). Root showed significantly higher arsenic concentrations compared to leaf (MD = 0.63; *p* = 0.044; 95% CI: 0.01–1.24), stem (MD = 1.12; *p* = 0.0001; 95% CI: 0.50–1.74), and grain (MD = 1.68; *p* = 0.0001; 95% CI: 1.06–2.29). The difference between leaf and stem was not significant (MD = 0.49; *p* = 0.163), while grain had significantly lower arsenic concentrations compared to leaf (MD = 1.05; *p* = 0.001; 95% CI: 0.44–1.67). The difference between stem and grain was also statistically significant at the 10% level (MD = 0.56; *p* = 0.089). Numerical results confirm that differences in arsenic concentrations (logAs) were greater and more consistent compared to copper (logCu), with a clear decreasing trend from soil to grain.

### 2.3. Ecological/Geogenic Impact

#### 2.3.1. Fe and Al in the Soil

**Figure 1:**
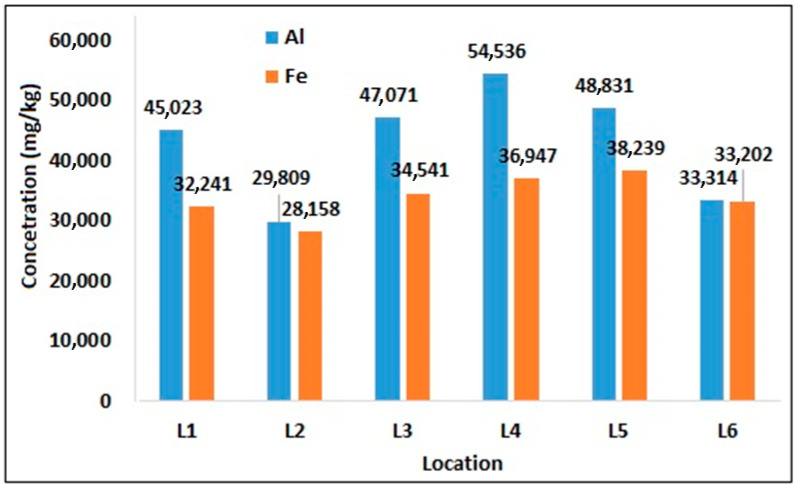
Concentration of Fe and Al in the soil.

The concentrations of matrix elements Fe and Al in soils across all locations indicate a stable geogenic composition, as their values fall within a relatively narrow range (Fe: 28,158–38,239 mg/kg; Al: 29,809–54,536 mg/kg) (Figure 1). This stability confirms that Fe and Al predominantly originate from natural geogenic sources and can be used as reliable reference elements in contamination assessment. At the reference location L6, Fe (33,037 mg/kg) and Al (33,280 mg/kg) concentrations confirm the natural background and the absence of anthropogenic influence. The concentrations of Cu and As (B_Cu_—20 mg/kg; B_As_—10 mg/kg) from location L6 were taken as background values, since this site is the most distant and therefore least affected by mining infrastructure. In contrast, locations L3 and L4 recorded the highest values of Al and Fe, reflecting a rich geogenic soil composition, but at the same time allowing clear identification of disproportionate Cu enrichment relative to them, originating from anthropogenic factors (mining and industrial activities). Locations L2 and L5 exhibited moderate Fe and Al concentrations, indicating the dominance of geogenic factors with minor anthropogenic contributions. These results confirm that Fe and Al represent the boundary between natural geogenic soil composition and anthropogenic enrichment with contaminants, thereby enabling clear differentiation of contamination sources.

Since the results in Figure 1 showed that Fe and Al have relatively similar values, the Fe_Al ratio was used for the calculation of ecological indices. The contamination index results for Cu clearly demonstrate pronounced differences between locations (Table 6). At the reference location L6, all index values (Igeo = −0.56; CF = 1.01; EF ≈ 1; Er = 5.07) indicate the absence of contamination. In contrast, locations L3 and L4 exhibited extremely high index values. At L4, CF = 28.43 and Igeo = 4.24 indicate very strong contamination, while EF values (17.36 for Al) confirm substantial Cu enrichment relative to the reference elements. The ecological risk (Er = 142.15) is classified as moderate but close to the threshold of high risk, pointing to significant anthropogenic influence from mining and industrial activities. A similar pattern was observed at L3 (CF = 18.84; Igeo = 3.65; EF up to 13.33; Er = 94.20), where index values were also well above threshold levels, indicating severe contamination and moderate risk. Location L1 exhibited moderate to strong contamination (Igeo = 2.85; CF = 10.84), with EF values of 8.02 (Al), indicating substantial Cu enrichment. The ecological risk at this location (Er = 54.22) falls within the category of moderate risk. Locations L2 and L5 showed lower index values (CF = 3.75 and 3.07; Igeo = 1.32 and 1.03), suggesting moderate contamination and low risk (Er = 18.75 and 15.36). These locations can be considered less impacted compared to L1, L3, and L4.

The contamination index results for As (Table 6) show that values are generally lower compared to Cu, but clear differences between locations are still evident. At the reference location L6, Igeo = −0.78 and CF = 0.87 indicate that As concentrations originate exclusively from geogenic sources, without anthropogenic enrichment, confirming its background status. A similar pattern was observed at L2 (Igeo = −0.60; CF = 0.99), where index values remain below threshold levels and indicate minimal risk (Er = 9.92). At L1, Igeo ≈ 0 and CF = 1.48 suggest slight As enrichment, but the EF value relative to Al (1.09) remains low, implying the dominance of geogenic factors. The ecological risk (Er = 14.76) is classified as low. Locations L3 and L5 exhibited moderate As enrichment. At L3, Igeo = 0.89 and CF = 2.79 indicate moderate contamination, while the EF value (1.97) confirms a combined influence of geogenic and anthropogenic factors. The ecological risk (Er = 27.88) remains within the low-to-moderate category. At L5, Igeo = 1.07 and CF = 3.16 indicate moderate contamination, with EF values (2.15) confirming slight anthropogenic enrichment. Er = 31.58 points to moderate risk. The most critical location was L4, where Igeo = 2.07 and CF = 6.30 exceeded the threshold for high contamination. EF values (3.85) indicate significant As enrichment relative to matrix elements, confirming anthropogenic contribution. The ecological risk (Er = 63.05) is classified as moderate but considerably higher than at other locations.

#### 2.3.2. Integrated Indices of Cu and As Contamination—RI, PLI

**Figure 2:**
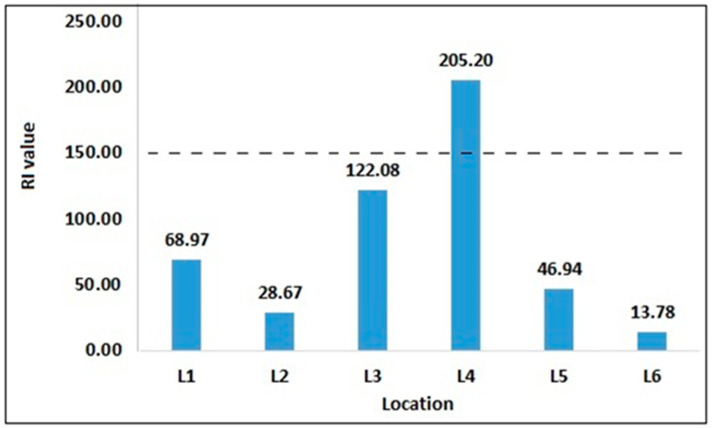
RI values of soils at six locations.

The analysis of the overall ecological risk (RI) showed that most locations fall into the low risk category (RI < 150), while only location L4 is classified as moderate risk (RI = 205.20), Figure 2. The lowest values were recorded at the reference location L6 (RI = 13.78), confirming its geogenic character and the absence of anthropogenic influence. These results indicate that metal contamination in soil has a localized ecological significance, with Cu contributing more to the overall risk compared to As, although no location exceeded the threshold for high risk (RI > 300).

The values of the PLI index clearly distinguish uncontaminated from contaminated sites. The reference location L6 (PLI = 0.94) confirms the natural geogenic character of the soil. Locations L2 and L5 show a moderate level of contamination (PLI of about 2–3), while L1 and L3 are considerably impacted (PLI = 4.00 and 7.25). The highest PLI value was recorded at L4 (PLI = 13.39), indicating extreme contamination and strong anthropogenic influence from mining and industrial activities. These results confirm that PLI is a reliable indicator of the overall soil contamination burden and clearly differentiates the boundary between geogenic and anthropogenic influences.

**Figure 3:**
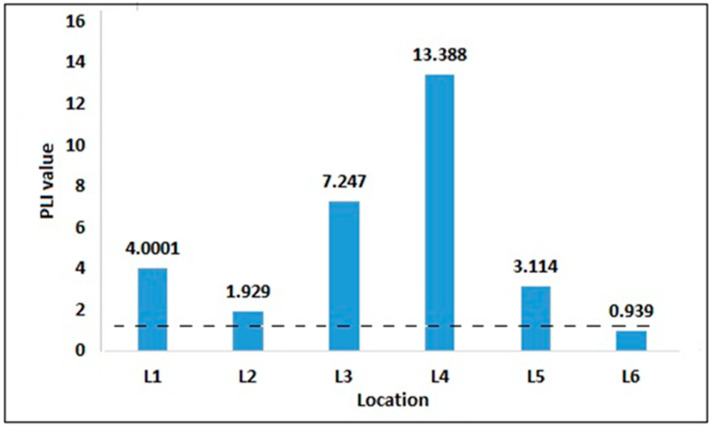
PLI soil values at six locations.

The values of the PLI index clearly separate uncontaminated from contaminated sites. The reference location L6 (PLI = 0.94, Figure 3) confirms the natural geogenic character of the soil. Locations L2 and L5 show a moderate level of contamination (PLI about 2–3), while L1 and L3 are considerably impacted (PLI = 4.00 and 7.25, respectively). The highest PLI value was recorded at L4 (PLI = 13.39), indicating extreme contamination and strong anthropogenic influence from mining and industrial activities. These results confirm that PLI is a reliable indicator of the overall soil contamination burden and clearly differentiates the boundary between geogenic and anthropogenic influences.

### 2.4. Biological Impact

**Figure 4:**
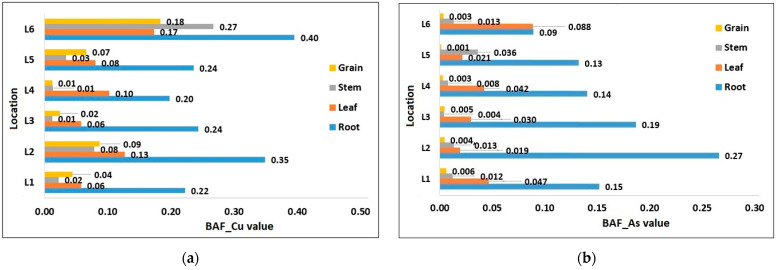
(**a**) BAF for Cu in parts of wheat at six locations. (**b**) BAF for As in parts of wheat at six locations.

The results of the bioaccumulation factor (BAF) indicate that wheat roots are the main accumulators of both Cu and As, with the highest values recorded at location L4 (BAF(Cu) = 0.19; BAF(As) = 0.14) (Figure 4a,b). These findings confirm that contamination from soil is primarily retained in the underground parts of the plant. Leaves showed moderate values, particularly for Cu at L4 (0.10), while stems and grains exhibited the lowest BAF values across all locations, indicating limited accumulation in the edible parts of wheat. Overall, the results clearly demonstrate that As remains predominantly bound in the roots, whereas Cu is translocated to aerial organs to a lesser extent.

**Figure 5:**
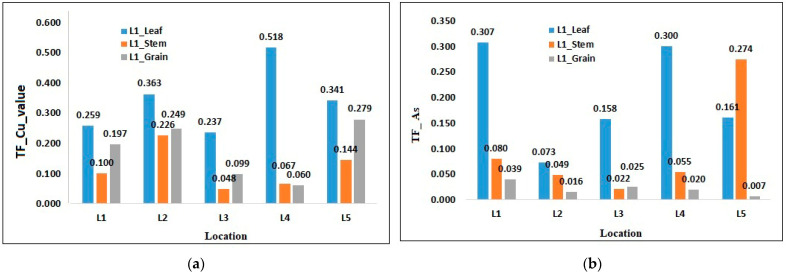
(**a**) TF for Cu in parts of wheat at six locations. (**b**). TA for As in parts of wheat at six locations.

The analysis of the translocation factor (TF) (Figure 5a,b) showed that values for Cu and As at all locations were below the threshold of TF = 1, indicating that both elements predominantly accumulate in the roots, while translocation to aerial organs is limited. The highest values for Cu (Figure 5a) were recorded in leaves at L4 (TF = 0.52), whereas values in grains remained low (TF < 0.30).

“For As, translocation was even weaker, with the highest values observed in leaves at L1 and L4 (TF ≈ 0.30) and in stems at L5 (TF = 0.27) (Figure 5b), while values in grains were minimal across all locations (TF < 0.05). These results indicate that the risk of Cu and As accumulation in the edible part of wheat is low, whereas vegetative organs (leaf, stem) may represent more significant sites of accumulation.

The translocation factor (TF) was considered but not included in the interpretation of results at location L6, since the obtained values were contradictory to other contamination and bioaccumulation indices and did not reflect the actual state of pollution at the Gornjane–L6 site. For the investigation at L6, TF proved to be an unreliable index.

### 2.5. Health Risk Assessment (HRA)

The HTA assessment was performed based on the values of the HQ and Hi indices. The health risk evaluation showed that the hazard quotient (HQ) values for copper (Cu) and arsenic (As) in wheat grain at all locations were above 1, indicating adverse effects on human health.

**Figure 6:**
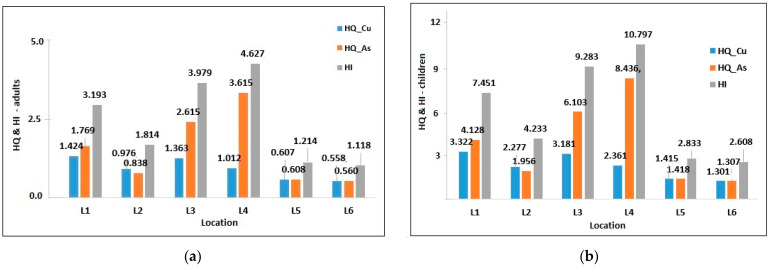
(**a**) HRA risk parameters for Cu and As in adult humans. (**b**) HRA risk parameters for Cu and As in children.

The health risk assessment indicates that the hazard quotient (HQ) values for copper (Cu) and arsenic (As) in wheat grain are above 1 at all sampling sites, suggesting a potential health risk. For adults (70 kg body weight, daily bread intake of 0.420 kg), as applied by Liu et al. (2023) [16], the combined hazard index (HI) reached the highest values at locations L4 (HI = 4.627) and L3 (HI = 3.979), Figure 6a, where arsenic was the dominant contributor (HQ_As = 2.615 and 3.615). At locations L1 and L2, HI values were 3.193 and 1.814, respectively, while the lowest values were recorded at L5 (HI = 1.214) and L6 (HI = 1.118). Although the values at L5 and L6 were lower, they still exceeded the threshold of 1, indicating that the risk cannot be considered negligible.

For children (15 kg body weight, daily bread intake of 0.210 kg), Figure 6b shows that the results are even more pronounced. HI values are considerably higher compared to adults, with maximum levels observed at L4 (HI = 10.797) and L3 (HI = 9.283). At locations L1 and L2, HI values are 7.451 and 4.233, respectively, while the lowest values are recorded at L5 (HI = 2.833) and L6 (HI = 2.608). These findings clearly demonstrate that children represent the most vulnerable population group, as their values are several times above the safety threshold, with arsenic being the dominant contributor.

Overall, the results indicate that arsenic is the primary risk factor in dietary exposure, while the contribution of copper is moderate but not negligible. At all locations, HI values exceeded the threshold of 1, suggesting the presence of chronic risk for both adults and children. Particularly concerning is the fact that values for children are several times higher, highlighting the urgent need for control measures to reduce contamination and limit exposure.

## 3. Discussion

The results of this research confirm that mining and metallurgical activities in Bor represent a long-term and dominant source of contamination for arable land and plant tissues. The highest concentrations of Cu and As were recorded in roots and leaves of wheat, while their values in the grain were lower, yet they still indicated a potential risk for human consumption. This distribution of metals is consistent with earlier findings in the region, where *Rosa* spp. and *Corylus* spp. showed pronounced accumulation of Cu and As in highly polluted environments [7,8].

The results of the one-way multivariate analysis of variance indicated that the Soil_Plant_Part factor had a statistically significant multivariate effect on the set of dependent variables. Post-hoc analysis revealed that soil and different parts of wheat differed significantly in copper and arsenic concentrations, with differences being more pronounced for As than for Cu. These findings suggest that the distribution of these elements within the plant is not homogeneous but depends on the soil and the specific wheat part. The obtained results cannot be directly compared with previous studies, since, to the best of the authors’ knowledge based on the review of relevant literature, earlier research has not addressed this topic in the same manner.

The geoaccumulation index (Igeo) and enrichment factor (EF) indicated moderate to very strong contamination, especially by Cu and As, confirming the anthropogenic nature of this pollution. Similar results were documented in studies that analyzed the impact of the copper smelter in Bor [1,4], as well as in other mining regions worldwide, such as Lanzhou in China and the Peruvian Andes, where integrated indices indicated a high environmental risk [10,11].

The analysis of the bioaccumulation index shows that roots and leaves are the main accumulators of Cu and As, while their translocation to the grain occurs to a lesser extent. Nevertheless, the presence of As in the grain, albeit in low concentrations, confirms a direct link between mining activities and food safety. Such findings are in accordance with studies by Filimon et al. (2021) and Gujre et al. (2021) [3,17], which indicated the accumulation of metals in edible parts of plants in mining regions.

The low translocation of Cu and As into wheat grain can be explained by plant exclusion mechanisms, in which metals are retained within roots or bound to cell walls, thereby reducing their transport to reproductive organs. These mechanisms represent an adaptive response by plants to heavy metal stress and have significant implications for food safety, as they limit the entry of toxic metals into the edible parts of the plant [13,14].

The distribution of Al, similar to Fe, also confirms its geogenic character and retention in the soil. Al does not pose a direct risk to food safety; however, its presence in the soil justifies its inclusion in ecological indices (Igeo, EF, and CF) to differentiate geogenic and anthropogenic sources of contamination.

Aggregate environmental risk (RI) and Site Pollution Index (PLI) clearly differentiate the most critical sites. Slatina-L4 and Oštrelj-L3 showed the highest values of Cu and As, while Zlot-L5 and Gornjane-L6 were relatively less polluted. This spatial distribution of risk indicates location-specific impacts of mining activities, which are in line with the findings by Serbul et al. (2017) and Kabala et al. (2020) [2,18]. A critical observation is that the problem should not be assessed solely based on average metal values but rather through spatial dynamics and local emission sources.

Our results indicate the long-term consequences of mining and metallurgical processes on agroecosystems. Reduced activity of soil enzymes in polluted sites confirms the degradation of ecosystem functions [5]. Thus, contamination should be considered not only a chemical problem but also a biological one, with implications for the productivity and sustainability of agricultural systems.

The TF results indicate a contradiction, as they show the highest As content in grain at the reference location L6, while the integrated analysis of indices clearly identifies L4 as the most contaminated zone. This discrepancy confirms that TF alone for location L6 is not a reliable indicator and should be interpreted in combination with other indices and the physiological mechanisms of bioaccumulation.

Although our results are in line with numerous international studies [19,20,21,22], they indicate the specificity of the Bor region, where the combination of mining activities, smelter emissions, and the historical legacy of pollution creates a unique ecological profile. This specificity requires locally adapted remediation strategies and monitoring because general approaches cannot adequately address the complexity of the problem.

**Scheme 1:**
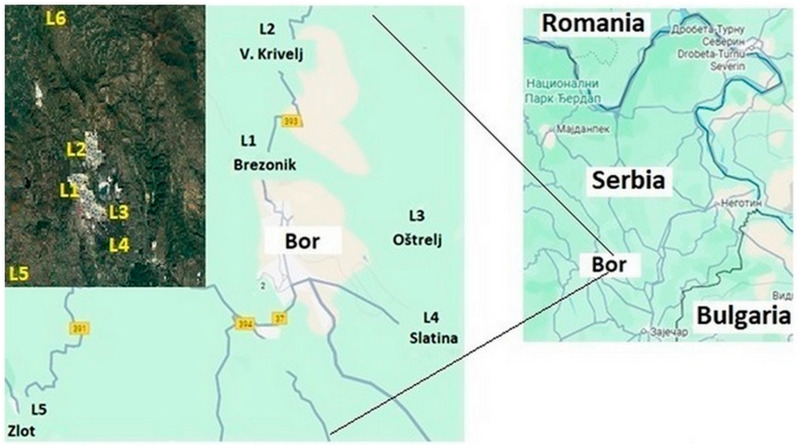
View of the map of the investigated locations.

Slatina-L4 stands out as the most critical location, with multiple exceedances of limit values for Cu and As. The values of Igeo, CF, Er, and RI confirm that Slatina falls within the high risk category. Oštrelj-L3, although slightly less affected, shows similar contamination patterns, with pronounced values of PLI and RI, which classify it among critical locations. A critical observation is that Slatina-L4 should be identified as the most endangered location, while Oštrelj-L3 ranks second in terms of pollution level, since these two sites are located close to each other, Scheme 1.

The results of metal bioaccumulation in wheat have direct implications for food safety, given that this cereal is still cultivated in the contaminated agroecosystem surrounding the Bor mining area.

### Practical Implications and Future Perspectives

The obtained results have significant implications for agroecological stability and food security in regions affected by mining and metallurgical activities. The identification of Slatina-L4 and Oštrelja-L3 as the most critical locations indicates the need for urgent intervention measures, including locally adapted remediation strategies and continuous monitoring. Such findings confirm that the problem of contamination is not uniform but spatially heterogeneous, which requires specific approaches to risk management [2,16].

Future perspectives include a more detailed investigation of the mechanisms of Cu and As translocation in plant tissues, with special emphasis on evaluating accumulation in edible parts of wheat. The development of biological and phytoremediation methods could contribute to reducing contamination and mitigating environmental consequences [7,8]. Filimon et al. 2021 [3] pointed out that the integration of ecotoxicological data into human health risk assessment models, along with monitoring contamination in the food chain, represents a key step toward understanding the cumulative effects on local communities.

Further research should contribute to the development of sustainable agricultural practices and land management strategies in mining regions to ensure long-term food security and preserve agroecological stability. A critical requirement is that remedial measures must be locally adapted and based on the spatial dynamics of contamination, as general approaches cannot adequately capture the complexity of the problem [10,11].

## 4. Materials and Methods

### 4.1. Sampling Locations

Sampling was carried out at six locations in the vicinity of the Zi Jin Bor mining and smelting complex, eastern Serbia: Brezonik, Veliki Krivelj—Banjica, Oštrelj, Slatina, Zlot, and Gornjane, as shown in Scheme 1. The locations were selected based on previous reports on the degree of pollution and spatial distribution of mining infrastructure. Gornjane-L6 was designated as a reference point due to its distance from the main sources of emissions and its position opposite to the wind rose directed at Slatina-4 and Oštrelj-3.

### 4.2. Sampling and Sample Preparation

At each location, soil samples were collected from a depth of up to 20 cm from plots under wheat (*Triticum aestivum* L.). Plant samples in the ripening phase were separated into root, stem, leaf, and grain. The plant material was cleaned, the roots were washed with distilled water, dried at room temperature, and homogenized. Soil samples were air-dried, sieved through a 2 mm sieve, and prepared for analysis. The sampling plan was developed in accordance with ISO 18400-101:2017 [23]. All procedures were carried out by accredited personnel from the Institute of Mining and Metallurgy Bor (IRM Bor), in compliance with ISO standards 18400-102:2017 [24], 18400-104:2018 [25], and 18400-105:2017 [26], as well as ISO 9001:2015 [27] and ISO/IEC 17025:2017 [28].

### 4.3. Experimental Part

Soil samples were digested in a microwave digestion system (PerkinElmer, Waltham, MA, USA) in the presence of HCl and HNO_3_ at a ratio of 3:1, following EPA 3051A [29]. Plant samples were digested using a combination of HNO_3_ and H_2_O_2_ at a ratio of 10:2. Quantitative analysis of Cu and As, as well as Fe and Al, was performed according to EPA 6020 [30] using ICP-MS (Agilent Technologies 7900, Santa Clara, CA, USA, with Agilent MassHunter software platform). The instrument was calibrated with standard solutions of known concentrations, and measurement accuracy was verified using certified reference materials, in accordance with ISO/IEC 17025:2017 [27].

### 4.4. Statistical Analysis and Ecological Risk Assessment

The aim of the study was to determine the significance of differences in copper and arsenic concentrations between soil and different parts of wheat. In line with this objective, the following hypothesis was formulated:

**H1:**  *There are statistically significant differences in copper and arsenic concentrations between soil and different parts of wheat (root, leaf, stem, and grain).*

To test the defined hypothesis, multivariate analysis of variance (MANOVA) was applied to examine differences in Cu and As concentrations between soils from six locations and various wheat parts (root, leaf, stem, and grain). Data were processed using IBM SPSS Statistics software, version 27. The dependent variables in the model were Cu and As concentrations, while the independent variables were soil from different locations (L1–L6) and wheat parts (Plant_Part: root, leaf, stem, and grain).

- For the assessment of Cu and As contamination in soil and wheat grown on that soil, the following indices were applied:
- Ecological risk assessment → Igeo, CF, EF, Er, RI, and PLI (for soil).
- Bioaccumulation and translocation → BFA and TR (for plants).
- Health risk → HRA (for humans, through the intake of contaminated plants).

Pollution indices (Igeo, CF, EF, Er, RI, and PLI) were determined for soil, whereas bioaccumulation (BFA) and translocation (TR) factors were calculated for plant samples. The coefficient of variation (CV) provides additional information on the variability of data in both soil and plant tissues. While the mentioned indices quantify the degree of contamination and metal transfer, the health risk assessment (HRA) links these results to the potential impact on human health.

The formulas of the applied indices, together with the threshold values, are presented in Table 7.

For the local background, values from location L6 were used, which was designated as a reference site since it is not directly influenced by mining and industrial activities. Accordingly, the following values were applied, as shown in Table 6: B_Cu_—20 mg/kg, B_As_—10 mg/kg, and BAlref—33,300 mg/kg.

It should be noted that the translocation factor (TF) was calculated, but for location L6 it was not used in the interpretation due to its inconsistency with other indices. For example, TF indicated the highest As content in grains at the reference location L6 (omitted in Figure 5a,b), whereas all other indices (Igeo, EF, CF, PLI, and BAF) identified L4 as the most problematic zone. This unreliability arises from the physiological mechanisms of plants, in which metals are retained in the roots and only partially translocated to the grains.

**Scheme 2:**
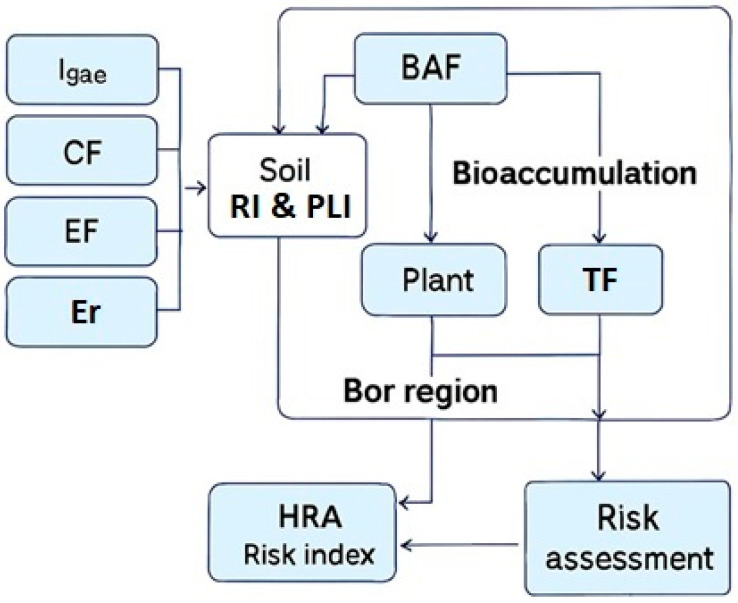
Conceptual flowchart illustrating the relationship between soil contamination indices (Igeo, EF, CF, Er, PLI, and RI), bioaccumulation in wheat (BAF and TF), and health risk assessment parameters (HRA: HQ, HI), applied in the Bor region.

The diagram reflects the methodological framework applied in this study, showing the progression from soil pollution to ecological and human health risk evaluation, Scheme 2.

The application of applied indices provides a comprehensive assessment of anthropogenic impact, identification of locations with the greatest environmental burden, and assessment of implications for agroecological stability and food safety [1,3,4,5,12].

## 5. Conclusions

This research confirms that mining and metallurgical activities in Bor represent a long-term and spatially differentiated source of contamination for arable land and wheat (*Triticum aestivum* L.). The highest concentrations of Cu and As in the soil were recorded in Slatina-L4 and Oštrelje-L3, where soil contamination indices (RI and PLI) indicated extreme values and were directly reflected in the roots and leaves of wheat. An integrated analysis of indices (PLI, RI, and HRA) showed that Slatina-L4 and Oštrelj-L3 are the most critical locations, while Gornjane-L6 can be considered a reference location with minimal risk. Such spatial differentiation emphasizes that the problem of contamination is not uniform but is influenced by the intensity of mining and metallurgical processes and the distance from mines and ore processing, as well as by the direction of the winds. The results for Al, which are included in the ecological indices (Igeo, EF, CF, and Er), confirm their predominantly geogenic character and indicate that these elements remain in the soil and roots, with limited translocation to aboveground organs and grain, thereby differing from anthropogenic contamination of Cu and As.

The root has proven to be the main reservoir of Cu and As, while the leaf shows significant translocation, especially at locations with high concentrations. Grain had the lowest values, but the presence of Cu and As, especially in Slatina-L4 and Oštrelje-L3, indicates a potential risk to human consumption. The ecological risk (Er) was several times more pronounced for As than for Cu, underscoring that As is a dominant factor in agroecosystem degradation and a key health risk.

In general, the results indicate that the assessment of soil contamination in combination with bioaccumulation and translocation factors in wheat provides a reliable basis for environmental and health risk assessment. The obtained research results demonstrated statistically significant differences in Cu and As concentrations between soils from six locations and different parts of wheat (root, leaf, stem, and grain). Arsenic stands out as the dominant health risk in the agroecosystem, while the concentration of Cu is lower, yet still present. These findings emphasize the need for continuous monitoring, locally adapted remediation strategies, and sustainable land management to ensure long-term food security and preserve agroecological stability in mining regions.

## Figures and Tables

**Table 1:** Descriptive statistics for Cu and As in soil at locations L1–L6.

L	Cu_mean	Cu_min	Cu_max	Cu_CV	As_mean	As_min	As_max	As_CV
L1	216.86 ± 8.50	210.85	222.87	3.92	14.76 ± 0.51	14.40	15.12	3.46
L2	75.00 ± 1.06	74.26	75.75	1.41	9.92 ± 0.41	9.63	10.21	4.13
L3	376.78 ± 4.24	373.78	379.78	1.13	27.88 ± 0.41	27.60	28.17	1.46
L4	568.60 ± 7.66	563.19	574.02	1.35	63.05 ± 0.81	62.47	63.62	1.29
L5	61.45 ± 1.17	60.62	62.28	1.90	31.58 ± 1.01	30.87	32.29	3.18
L6	20.28 ± 0.01	20.28	20.29	0.03	8.71 ± 0.14	8.61	8.81	1.63

**Table 2:** Descriptive statistics for Cu and As in wheat at locations L1–L6.

L	Sl_Type	Cu_mean	Cu_min	Cu_max	Cu_CV	As_mean	As_min	As_max	As_CV
L1	Grain	9.49 ± 2.11	8.00	10.99	22.26	0.09 ± 0.07	0.04	0.14	76.24
L1	Stem	4.81 ± 1.15	4.00	5.63	23.91	0.18 ± 0.06	0.14	0.22	31.09
L1	Leaf	12.48 ± 0.50	12.12	12.83	4.01	0.69 ± 0.02	0.67	0.70	2.77
L1	Root	48.15 ± 31.88	25.61	70.69	66.20	2.24 ± 1.71	1.03	3.45	76.38
L2	Grain	6.51 ± 0.49	6.16	6.85	7.57	0.04 ± 0.00	0.04	0.05	11.68
L2	Stem	5.90 ± 0.69	5.41	6.39	11.73	0.13 ± 0.12	0.04	0.22	95.12
L2	Leaf	9.50 ± 1.35	8.55	10.46	14.17	0.19 ± 0.03	0.17	0.21	14.90
L2	Root	26.16 ± 0.94	25.50	26.83	3.59	2.64 ± 0.27	2.45	2.83	10.10
L3	Grain	9.09 ± 0.00	9.09	9.09	0.04	0.13 ± 0.01	0.13	0.13	4.16
L3	Stem	4.39 ± 1.70	3.20	5.59	38.60	0.11 ± 0.00	0.11	0.12	3.36
L3	Leaf	21.69 ± 2.00	20.28	23.10	9.21	0.82 ± 0.06	0.78	0.87	7.53
L3	Root	91.71 ± 25.86	73.43	110.00	28.20	5.21 ± 2.30	3.58	6.83	44.21
L4	Grain	6.75 ± 0.92	6.10	7.39	13.57	0.18 ± 0.01	0.17	0.19	6.02
L4	Stem	7.55 ± 1.01	6.84	8.27	13.32	0.48 ± 0.04	0.46	0.51	8.53
L4	Leaf	58.31 ± 28.07	38.46	78.15	48.14	2.66 ± 0.55	2.27	3.05	20.78
L4	Root	112.47 ± 1.77	111.21	113.72	1.58	8.86 ± 0.13	8.77	8.95	1.41
L5	Grain	4.04 ± 0.02	4.03	4.06	0.41	0.03 ± 0.00	0.03	0.03	16.11
L5	Stem	2.08 ± 0.08	2.03	2.14	3.63	1.15 ± 0.22	0.99	1.30	19.19
L5	Leaf	4.94 ± 1.85	3.63	6.25	37.40	0.67 ± 0.01	0.66	0.68	2.02
L5	Root	14.47 ± 1.31	13.54	15.40	9.08	4.18 ± 0.55	3.79	4.57	13.25
L6	Grain	3.72 ± 0.10	3.65	3.79	2.63	0.03 ± 0.00	0.03	0.03	0.00
L6	Stem	5.42 ± 5.32	1.66	9.18	98.13	0.12 ± 0.01	0.11	0.12	7.35
L6	Leaf	3.51 ± 0.62	3.07	3.95	17.55	0.77 ± 0.04	0.74	0.80	5.39
L6	Root	8.02 ± 2.80	6.04	10.00	34.90	0.78 ± 0.06	0.74	0.82	7.26

**Table 3:** Multivariate tests.

Multivariate Tests ^a^							
Effect		Value	F	Hypothesis df	Error df	Sig.	Partial Eta Squared
Intercept	Pillai’s Trace	0.943	196.881 ^b^	2.000	24.000	0.000	0.943
	Wilks’ Lambda	0.057	196.881 ^b^	2.000	24.000	0.000	0.943
	Hotelling’s Trace	16.407	196.881 ^b^	2.000	24.000	0.000	0.943
	Roy’s Largest Root	16.407	196.881 ^b^	2.000	24.000	0.000	0.943
Soil_Plant_Part	Pillai’s Trace	1.073	7.231	8.000	50.000	0.000	0.536
	Wilks’ Lambda	0.102	12.824 ^b^	8.000	48.000	0.000	0.681
	Hotelling’s Trace	7.127	20.489	8.000	46.000	0.000	0.781
	Roy’s Largest Root	6.877	42.982 ^c^	4.000	25.000	0.000	0.873

^a^ Design: Intercept + Soil_Plant_Part; ^b^ Exact statistic; ^c^ The statistic is an upper bound on F that yields a lower bound on the significance level.

**Table 4:** Tests of between-subjects effects.

Tests of Between-Subjects Effects							
Source	Dependent Variable	Type III Sum of Squares	df	Mean Square	F	Sig.	Partial Eta Squared
Corrected Model	logCu	8.323 ^a^	4	2.081	13.618	0.000	0.685
	logAs	22.642 ^b^	4	5.660	42.898	0.000	0.873
Intercept	logCu	45.668	1	45.668	298.882	0.000	0.923
	logAs	0.029	1	0.029	0.216	0.646	0.009
Soil_Plant_Part	logCu	8.323	4	2.081	13.618	0.000	0.685
	logAs	22.642	4	5.660	42.898	0.000	0.873
Error	logCu	3.820	25	0.153			
	logAs	3.299	25	0.132			
Total	logCu	57.811	30				
	logAs	25.969	30				
Corrected Total	logCu	12.143	29				
	logAs	25.941	29				

^a^ R squared = 0.685 (adjusted R squared = 0.635); ^b^ R squared = 0.873 (adjusted R squared = 0.852).

**Table 5:** Multiple comparisons (Tukey HSD).

Dependent Variable	(I) Plant_Part	(J) Plant_Part	Mean Difference (I–J)	Std. Error	Sig.	95% Confidence Interval	
						Lower Bound	Upper Bound
logCu	Soil	Root	0.5765	0.22568	0.110	−0.0863	1.2393
		Leaf	1.0371 *	0.22568	0.001	0.3743	1.6999
		Stem	1.4351 *	0.22568	0.000	0.7723	2.0979
		Grain	1.3138 *	0.22568	0.000	0.6510	1.9766
	Root	Soil	−0.5765	0.22568	0.110	−1.2393	0.0863
		Leaf	0.4606	0.22568	0.277	−0.2022	1.1234
		Stem	0.8586 *	0.22568	0.007	0.1958	1.5214
		Grain	0.7373 *	0.22568	0.024	0.0745	1.4001
	Leaf	Soil	−1.0371 *	0.22568	0.001	−1.6999	−0.3743
		Root	−0.4606	0.22568	0.277	−1.1234	0.2022
		Stem	0.3980	0.22568	0.416	−0.2648	1.0608
		Grain	0.2767	0.22568	0.737	−0.3861	0.9395
	Stem	Soil	−1.4351 *	0.22568	0.000	−2.0979	−0.7723
		Root	−0.8586 *	0.22568	0.007	−1.5214	−0.1958
		Leaf	−0.3980	0.22568	0.416	−1.0608	0.2648
		Grain	−0.1214	0.22568	0.982	−0.7842	0.5414
	Grain	Soil	−1.3138 *	0.22568	0.000	−1.9766	−0.6510
		Root	−0.7373 *	0.22568	0.024	−1.4001	−0.0745
		Leaf	−0.2767	0.22568	0.737	−0.9395	0.3861
		Stem	0.1214	0.22568	0.982	−0.5414	0.7842
logAs	Soil	Root	0.8173 *	0.20972	0.005	0.2014	1.4332
		Leaf	1.4451 *	0.20972	0.000	0.8291	2.0610
		Stem	1.9374 *	0.20972	0.000	1.3215	2.5533
		Grain	2.4963 *	0.20972	0.000	1.8803	3.1122
	Root	Soil	−0.8173 *	0.20972	0.005	−1.4332	−0.2014
		Leaf	−0.6278 *	0.20972	0.044	0.0119	1.2437
		Stem	1.1201 *	0.20972	0.000	0.5042	1.7360
		Grain	1.6790 *	0.20972	0.000	1.0630	2.2949
	Leaf	Soil	−1.4451 *	0.20972	0.000	−2.0610	−0.8291
		Root	−0.6278 *	0.20972	0.044	−1.2437	−0.0119
		Stem	0.4923	0.20972	0.163	−0.1236	1.1082
		Grain	1.0512 *	0.20972	0.000	0.4352	1.6671
	Stem	Soil	−1.9374 *	0.20972	0.000	−2.5533	−1.3215
		Root	−1.1201 *	0.20972	0.000	−1.7360	−0.5042
		Leaf	−0.4923	0.20972	0.163	−1.1082	0.1236
		Grain	0.5589	0.20972	0.089	−0.0571	1.1748
	Grain	Soil	−2.4963 *	0.20972	0.000	−3.1122	−1.8803
		Root	−1.6790 *	0.20972	0.000	−2.2949	−1.0630
		Leaf	−1.0512 *	0.20972	0.000	−1.6671	−0.4352
		Stem	−0.5589	0.20972	0.089	−1.1748	0.0571

* The mean difference is significant at the 0.05 level.

**Table 6:** (**a**) Results of Cu contamination index in soil, (**b**) Results of As contamination index in soil.

(**a**)				
**Location**	**Igeo**	**CF**	**EF_Al**	**Er**
L1	2.85	10.84	8.02	54.22
L2	1.32	3.75	4.19	18.75
L3	3.65	18.84	13.33	94.20
L4	4.24	28.43	17.36	142.15
L5	1.03	3.07	2.10	15.36
L6	−0.56	1.01	1.01	5.07
(**b**)				
**Location**	**Igeo**	**CF**	**EF_Al**	**Er**
L1	−0.02	1.48	1.09	14.76
L2	−0.60	0.99	1.11	9.92
L3	0.89	2.79	1.97	27.88
L4	2.07	6.30	3.85	63.05
L5	1.07	3.16	2.15	31.58
L6	−0.78	0.87	0.87	8.71

Note: EF—for As using Al as a reference element. The real values for CF and EF are close to unity, but a rounded background value of 10 was used for As.

**Table 7:** Formulas of the indices applied to arable soil and wheat parts, along with the corresponding threshold values.

Index *	Formula	Meaning in the Formula	Threshold Values	Ref.
CV	(SD/Mean) × 100	SD—standard deviation	<20% low, 20–50% moderate >50% high	[31]
Igeo	log_2_ (C_n_/(1.5 × B_n_))	C_n_—concentration of the element (sample) B_n_—background concentration of the element 1,5—correction due to lithological effects	≤0 uncontaminated 1–2 moderately >5 extremely contaminated	[32]
EF	(C_n_/B_ref_)_sample_/(C_n_/B_ref_)_background_	C_n_—concentration of the element B_ref_—concentration of the reference element (Al, Fe)	≤2 geogenic >40 extremely high	[33]
CF	C_n_/B_n_	C_n_—concentration of the element (sample) B_n_—background concentration of the element	<1 low >6 very high	[33]
PLI	(CF_1_ × CF_2_ × … × CF_n_)^(1/n)^	CF_n_—concentration factor for each element	=1 basic, >1 polluted	[34]
Er	Tr × CF	Tr—toxic response factor (for As = 10, Cu = 5) CF—contamination factor for each element	<40 low >320 very high	[33]
RI	ΣEr	Er—ecological risk factor	<150 low >600 very high	[33]
BAF	C_plant_/C_soil_	C_plant_—element concentration in plant C_soil_—element concentration in soil	<1 no accumulation ≥1 accumulation	[35]
TF	C_shoot_/C_root_	C_shoot_—element concentration in plant organs C_root_—element concentration in root	<1 root >1 translocation	[35]
HRA	HQ = ADI/RfD = (C_n_ × D/B)/RfD HI = ΣHQ	ADI—average daily intake, mg kg^−1^day^−1^ C_n_—metal concentration in grain, mg/kg D—daily wheat intake, kg B—average body weight, kg RfD—chronic toxicity reference dose, mg kg^−1^day^−1^ (for Cu = 0.04, As = 0.0003)	HI < 1 no health risk HI ≥ 1 adverse noncarcinogenic effects on health	[4,16]

* Note: CV—coefficient of variation, Igeo—geoaccumulation index, EF—enrichment factor, CF—contamination factor, PLI—pollution load index, Er—ecological risk factor, RI—potential ecological risk index, BAF—bioaccumulation factor, TF—transclocation factor, HRA—health risk assessment, HQ—hazard quotient, HI—hazard index.

## Data Availability

The datasets generated and/or analyzed during the current study are available from the corresponding author upon reasonable request.

## Abbreviations

The following abbreviations are used in this manuscript:

CV	Coefficient of Variation
Igeo	Geoaccumulation Index
EF	Enrichment Factor
CF	Contamination Factor
Er	Ecological Risk
PLI	Pollution Load Index
IR	Risk Index
BAF	Bioaccumulation Factor
HRA	Health Risk Assessment
HQ	Hazard Quotient
HI	Hazard Index
